# Characterization of a Novel Quorum Quencher *Acinetobacter schindleri* Strain XJ-10: AHL Degradation Capability, Metabolic Pathways and Its Role in Soft Rot Disease Biocontrol

**DOI:** 10.3390/plants15162439

**Published:** 2026-08-11

**Authors:** Xiaofang Luo, Hui Liu, Zhihao Wen, Wen-Juan Chen, Xinghui Fan, Mohamed A. Ghorab, Shaohua Chen, Yonglin Liao

**Affiliations:** 1 Guangdong Province Key Laboratory of Microbial Signals and Disease Control, Integrative Microbiology Research Centre, South China Agricultural University, Guangzhou 510642, China; 2 Guangdong Provincial Key Laboratory of High Technology for Plant Protection, Institute of Plant Protection, Guangdong Academy of Agricultural Sciences, Guangzhou 510640, China; 3 Wildlife Toxicology Laboratory, Department of Animal Science, Institute for Integrative Toxicology (IIT), Michigan State University, East Lansing, MI 48824, USA

**Keywords:** biocontrol, quorum sensing, quorum quenching, AHL, plant diseases

## Abstract

Quorum sensing (QS) is critically involved in mediating microbial interactions and serves as a central regulatory mechanism in bacterial pathogenesis. As an emerging countermeasure, quorum quenching (QQ) suppresses QS-regulated virulence through enzymatic or chemical disruption of signal systems. *N*-acyl homoserine lactone (AHL), an evolutionarily conserved QS signal, coordinates the pathogenicity of multiple plant pathogens, particularly *Dickeya zeae*, which causes soft rot disease in various crops and leads to substantial agricultural losses. In this study, the QQ strain *Acinetobacter schindleri* XJ-10 was evaluated for its capacity to degrade AHL and attenuate the pathogenicity of *D. zeae* EC1 in host plants. Notably, strain XJ-10 exhibited efficient AHL degradation at 0.2 mmol/L within 24 h, achieving a degradation efficiency of 98.80%. Subsequently, gas chromatography–mass spectrometry (GC-MS) analysis identified *N*-hexanoyl-L-homoserine lactone and propanamide as key intermediates during AHL degradation, confirming complete mineralization to CO_2_ and H_2_O. Based on the structural characterization of AHL and its intermediates, the metabolic pathway within strain XJ-10 was proposed. The degradation pathway initiates with the hydrolysis of the ester ring of *N*-hexanoyl-L-homoserine lactone, generating *N*-hexanoyl-L-homoserine. Subsequent carbon–nitrogen bond scission is predicted to yield *N*-cyclohexyl-propanamide, which is further catabolized to produce hexanamide and propanamide. Furthermore, strain XJ-10 exhibited biocontrol activity against soft rot disease affecting potato (*Solanum tuberosum*), radish (*Raphanus sativus*), and Chinese cabbage (*Brassica rapa* subsp. *pekinensis*), as its crude enzyme extract effectively reduced disease incidence and severity *in planta*. While strain XJ-10 showed no detectable acylase activity, it exhibited significant degradation activity against AHL, suggesting a distinct QQ mechanism. Collectively, these findings broaden the scope of QQ-based biocontrol strategies and enhance mechanistic insights into managing bacterial diseases through QS modulation.

## 1. Introduction

Quorum Sensing (QS) represents a density-dependent regulatory mechanism governing microbial population-level behaviors through extracellular signaling molecules [1,2,3,4]. This system activates target gene expression upon reaching autoinducer concentration thresholds [5,6]. As an intercellular communication system that alters microbial interactions [7,8,9], QS orchestrates critical biological processes: virulence expression, biofilm assembly, sporulation initiation, motility regulation, and antimicrobial resistance [10,11]. Moreover, QS coordinates population-wide behavioral adaptation to density fluctuations and community dynamics via collective gene expression regulation [12,13,14]. At the molecular level, bacterial cells synthesize diffusible signaling molecules that traverse cell membranes into the extracellular milieu. Simultaneously, bacteria detect signaling molecules released by neighboring cells [15,16]. Subsequently, density-dependent accumulation of signaling molecules activates receptor-mediated signaling cascades upon reaching critical thresholds, triggering population-wide transcriptional reprogramming [17,18]. This process activates a series of signaling cascades and gene expression programs, regulating microbial material and energy metabolism while enhancing resilience [19,20]. However, the amplification of population-level responses inherent to QS can disrupt microbial homeostasis, leading to challenges in wastewater treatment efficiency, clinical pathogen persistence, and agricultural microbiome stability [21,22,23]. Notably, AHL-dependent QS systems are critically involved in regulating the virulence of *Dickeya zeae*, a notorious phytopathogen causing soft rot disease in various crops.

*Dickeya zeae* (formerly *Erwinia chrysanthemi* pv. *zeae*) is a plant-pathogenic bacterium causing soft rot in both monocotyledonous and dicotyledonous plants. This species demonstrates a broad host range, including economically important crops such as bananas, rice, maize, Ipomoea batatas, potatoes, etc. [24,25]. The pathogen induces characteristic tissue maceration through enzymatic degradation, manifesting as structural collapse and putrid odor emission, resulting in significant economic losses across global agricultural systems [26]. Central to its virulence is a QS regulatory system mediated by the LuxI homolog ExpI [24]. This synthase produces *N*-acyl homoserine lactones (AHLs) that coordinate quorum-dependent activation of plant cell wall-degrading enzyme (CWDE) gene clusters, culminating in enzymatic tissue maceration during high-cell-density pathogenesis [27,28,29]. Conventional management of *D. zeae*-induced soft rot remains challenging due to its exceptionally broad host range and the emergence of antibiotic-resistant pathogen strains, while no single chemical agent has proven completely effective. Therefore, there is an urgent need to develop alternative approaches for effective disease management.

As Gram-negative-specific QS signals, AHLs are characterized by a conserved structure consisting of a lactone ring connected to a variable acyl chain via an amide bond [30,31]. *N*-(3-oxohexanoyl)-L-homoserine lactone (OHHL) and *N*-(3-oxododecanoyl)-L-homoserine lactone (OdDHL) are classified as AHLs, differing primarily in their acyl side chain lengths, which influence their signaling specificities and functional outcomes [32,33]. This structure-function relationship drives species-specific behaviors. *D. zeae* EC1 employs short-chain OHHL to activate CWDE-mediated host invasion [24], while the long acyl chain of OdDHL enables *Pseudomonas aeruginosa* to regulate biofilm maturation and virulence expression through enhanced membrane permeability [32,34].

Quorum quenching (QQ) is a deliberate approach to disrupt the QS process [35,36,37]. QQ disrupts microbial communication through two principal modalities: biocatalytic degradation and molecular structural alteration of autoinducers [38,39]. This dual mechanism effectively inhibits the activation of pathogenicity-related genes mediated by QS pathways [40,41,42]. QQ functions through four primary mechanisms: suppression of autoinducer synthase activity, enzymatic degradation of AHLs, competitive inhibition of LuxR-type receptor binding and interference with phosphorelay signal transduction pathways [43]. Phytopathogens use QS networks to synchronize infection progression by coordinating virulence factor production during host tissue colonization [44,45]. Natural-product-derived QQ agents specifically disrupt microbial communication systems, providing environmentally sustainable solutions for managing persistent crop diseases, such as soft rot. This strategy provides dual agricultural benefits: it reduces the development of chemical pesticide resistance and maintains a balanced functional microbiome within agroecosystems [46]. QQ-based approaches demonstrate significant potential in counteracting agrochemical resistance by precisely disrupting pathogenicity-regulatory mechanisms.

Current strategies for controlling phytobacterial diseases primarily rely on chemical bactericides and resistant cultivars. However, these methods pose significant risks, including ecotoxicity, the development of pathogen resistance, and impacts on non-target organisms [47]. The global antimicrobial resistance crisis, driven by antibiotic overuse, highlights the urgent requirement for novel control strategies [48,49,50,51]. Existing biological control approaches against soft rot pathogens comprise four main strategies: bacterial antagonist application, phage therapy, QQ implementation and plant systemic resistance induction [52,53]. Unlike conventional antimicrobials, QQ technology specifically targets virulence determinants via signal molecule interference instead of microbial lethality, thus reducing selection pressure for resistance emergence [36,54,55,56]. While current research on QQ has predominantly focused on *Pseudomonas* and *Bacillus* species as biocontrol agents [31], this study reveals the potential of *Acinetobacter* in disrupting bacterial communication. Specifically, the *Acinetobacter schindleri* strain XJ-10 exhibits rapid AHL degradation kinetics and demonstrates significant efficacy in suppressing AHL-mediated phytopathogenic infections. This finding expands the taxonomic scope of QQ-competent microbes and provides a novel biocontrol alternative for plant disease management.

In this study, *A. schindleri* strain XJ-10 was identified and characterized as a novel QQ agent, and its AHL degradation mechanisms and metabolic products were systematically investigated. Additionally, the efficacy of strain XJ-10 and its crude enzyme against bacterial soft rot pathogens was assessed in vitro. This assessment aimed to provide a biocontrol strategy for managing AHL-dependent phytopathogens via targeted QQ interventions.

## 2. Materials and Methods

### 2.1. Strains, Media, Plants and Chemicals

*Dickeya zeae* EC1, *Bacillus thuringiensis* subsp. *israelensis* B23, and the reporter strain *Agrobacterium tumefaciens* NT1 were obtained from the Integrative Microbiology Research Centre at South China Agricultural University in Guangzhou, China.

*D. zeae* EC1 (potato soft rot pathogen), *B. thuringiensis* B23, and *A. tumefaciens* NT1 were cultured in Luria–Bertani (LB) medium (5.0 g/L yeast extract, 10.0 g/L tryptone, 10.0 g/L NaCl) at 30 °C with shaking at 200 rpm, while *Escherichia coli* DH5α was grown in LB medium at 37 °C. Two defined media were autoclaved (121 °C, 20 min) for strain XJ-10 assays. The minimal medium (MM) comprises (NH_4_)_2_SO_4_ (2.0 g), MgSO_4_·7H_2_O (0.2 g), CaCl_2_ (0.01 g), FeSO_4_ (0.005 g), MnCl_2_ (0.002 g), K_2_HPO_4_ (10.5 g), KH_2_PO_4_ (4.5 g), mannitol (2.0 g), and glycerol (2.0 g). The mineral salt medium (MSM) consists of (NH_4_)_2_SO_4_ (2.0 g), Na_2_HPO_4_·12H_2_O (1.5 g), KH_2_PO_4_ (1.5 g), MgSO_4_·7H_2_O (0.2 g), CaCl_2_·2H_2_O (0.01 g), and FeSO_4_·7H_2_O (0.001 g) dissolved in 1 L of deionized water (pH 6.5). Both media were used to evaluate *N*-acyl homoserine lactone (AHL) degradation efficiency and metabolite identification by strain XJ-10.

Healthy cabbage (*Brassica rapa* subsp. *pekinensis*), radish (*Raphanus sativus*) and potato (*Solanum tuberosum*) plants were purchased from a local market in Guangzhou, China, and used for experimental assays. The following materials were sourced commercially: AHL signaling molecules: *N*-(3-oxododecanoyl)-L-homoserine lactone (OdDHL) from Shanghai Youde Chemical Technology Co., Ltd. (Shanghai, China); X-gal (5-bromo-4-chloro-3-indolyl-β-d-galactoside) and antibiotics for susceptibility tests: Ampicillin (Amp), Chloramphenicol (Cm), Streptomycin (Str), Gentamicin (Gen), Kanamycin (Kan), and Tetracycline (Tc) from Guangzhou Qixiang and Hua Qisheng Co., Ltd. (Guangzhou, China).

### 2.2. Isolation and Screening of AHL-Degrading Bacteria

Soil specimens were collected from agricultural land (43°40′06″ N, 87°19′15″ E) in the Xinjiang Uygur Autonomous Region, China, in October 2014 and transported to the laboratory in sterile polyethylene bags kept at 4 °C. Following the enrichment culture protocol described by [57], 5 g of homogenized soil was inoculated into MSM for the screening of AHL-degrading bacteria. Bacterial isolation was initially performed using the spread plate method, and pure colonies were obtained through repeated streaking on LB agar plates [5,58,59,60]. Single isolates were subsequently subjected to AHL degradation activity assays as previously reported.

The biosensor strain *A. tumefaciens* NT1 was utilized for the colorimetric detection of OdDHL degradation activity through a blue chromogenic reaction [61,62]. Experimental controls included *E. coli* DH5α as a negative control (non-degrading strain), *B. thuringiensis* B23 as a positive control (validated OdDHL degrader), and uninoculated sterile medium as a blank control [63].

This detection system operates through AHL-mediated induction of LacZ-encoded β-galactosidase activity, which catalyzes X-gal hydrolysis to produce a blue chromogenic product [23,61]. The chromogenic response exhibited concentration dependency based on OdDHL diffusion kinetics: elevated residual OdDHL levels promoted longer diffusion distances from the inoculation site, corresponding to expanded blue halo radii on MM plates. Comparative quantification revealed that test samples displaying halo diameters comparable to uninoculated controls (CKs) indicated negligible OdDHL degradation. Conversely, significant reductions in halo dimensions or complete absence of coloration demonstrated effective OdDHL catabolism. Through this bioassay, strain XJ-10 was identified as exhibiting superior OdDHL degradation capacity, manifesting smaller halo areas compared to CKs.

### 2.3. Identification of Strain XJ-10

The bacterial strain XJ-10 underwent taxonomic characterization based on morphological, physiological, and 16S rDNA phylogenetic analysis. Strain XJ-10 was seeded onto LB agar medium and maintained under 30 °C conditions for 24 h. Morphological characterization of microbial colonies was conducted using light microscopy (BH-2 Olympus, Tokyo, Japan), while cellular ultrastructure analysis employed scanning electron microscopy (Hitachi, Tokyo, Japan). Bacterial whole-genome DNA isolation was performed using the EasyPure Bacterial Genomic DNA Kit (Beijing, China). PCR amplification of the 16S rDNA gene employed universal bacterial primers 27F (5′-AGAGTTTGATCCTGGCTCA-3′) and 1492R (5′-GGTTACCTTGTTACGACTT-3′) designed according to established protocols. PCR conditions were: initial denaturation at 94 °C for 5 min and 30 s, annealing at 55 °C for 30 s, extension at 72 °C for 1 min and 30 s, and a final cycle of extension at 72 °C for 5 min. Purified PCR products were sequenced by Invitrogen Science and Technology Co., Ltd. in Shanghai, China. Sequence homology was determined through BLAST analysis against the NCBI database (https://blast.ncbi.nlm.nih.gov/Blast.cgi, accessed on 30 July 2026), with phylogenetic reconstruction performed in MEGA 6.0 using the neighbor-joining algorithm [28].

### 2.4. Antibiotic Susceptibility Test

To evaluate the biocontrol potential of strain XJ-10, antibiotic tolerance was assessed using gradient concentration assays. Strain XJ-10 was activated at 30 °C for 24 h. Single colonies were inoculated in 5 mL of LB medium and incubated with different concentrations of various antibiotics overnight at 30 °C and 200 rpm. The final concentration gradients of five antibiotics (Amp, Cm, Str, Gen, Kan, and Tc) were 5, 10, 20, 50, 100, 150, 200, 250, 300, 350, and 400 μg/mL. Ten-fold concentration gradients (5–400 μg/mL) were tested in triplicate. Bacterial growth under antibiotic stress was monitored by spectrophotometric measurement (OD_600_) after 24 h of incubation.

### 2.5. Biodegradation Assays

To investigate the correlation between the degradation kinetics of OdDHL and the growth of strain XJ-10, residual OdDHL concentrations were quantified using high-performance liquid chromatography (HPLC) at designated time intervals. Individual colonies were aseptically transferred to LB broth for primary cultivation until they reached the logarithmic phase. Subsequently, the bacterial culture was transferred to MSM supplemented with OdDHL, with the initial cell density adjusted by OD_600_ and incubated at 30 °C with agitation at 200 rpm. Samples were collected periodically, with triplicate biological replicates taken at each time point. Following a 10-min centrifugation at 4000 rpm, the liquid fraction was washed with acetonitrile and subsequently filtered through an organic membrane. OD_600_ measurements (UV mini-1240 UV-Vis spectrophotometer, Shimadzu, Kyoto, Japan) and HPLC analyses (Alliance 2695, Waters, Milford, MA, USA) were conducted to assess bacterial growth and OdDHL degradation, respectively. All degradation tests were performed in triplicate.

### 2.6. Identification of AHL Degradation Metabolites by GC–MS

Samples were periodically collected and processed as previously described. The degradation products of OdDHL were analyzed using gas chromatography–mass spectrometry (GC-MS) with an Agilent 6890N/5975 system (Agilent Technologies, Santa Clara, CA, USA), employing uninoculated OdDHL-containing samples as controls. Chromatographic separation was conducted on an Agilent HP-5MS capillary column (30 m × 250 μm × 0.25 μm) with high-purity helium as the carrier gas at a flow rate of 1 mL/min. The inlet and transfer line temperatures were maintained at 200 °C and 280 °C, respectively. Mass spectrometry was performed in electron ionization (EI) mode at 70 eV, with the ion source and quadrupole temperatures set at 230 °C and 150 °C, respectively. Retention times and mass spectra were compared against NIST library standards for compound identification.

### 2.7. Antagonistic Test of Strain XJ-10

The antagonistic activity of XJ-10 against *Dickeya zeae* EC1 was quantified through agar-well diffusion assays following [56]. LB agar plates pre-inoculated with *D. zeae* EC1 were prepared, and uniform wells were created using a sterile cork borer. Each well received 20 μL of the following test solutions: XJ-10 cell suspension, XJ-10 metabolite extract, acetonitrile, and sterile water, with triplicate biological replicates per treatment. After 24 h incubation at 30 °C, the antagonistic effects were determined by observing clear inhibition zones around the wells.

### 2.8. Biocontrol Test of Acinetobacter schindleri XJ-10 Against Dickeya zeae

To evaluate the bioprophylactic efficacy of strain XJ-10 against AHL-dependent pathogenic bacteria, dip inoculation experiments were conducted following established methodologies [24,56,63]. Bacterial strains, including XJ-10, EC1, DH5α, and B23, were initially cultured on solid media at 30 °C for 24 h. Individual colonies were then inoculated into LB medium and incubated aerobically at 30 °C with 200 rpm agitation until reaching stationary phase (OD_600_ > 2.0).

Fresh market-sourced radishes and potato tubers were prepared as experimental substrates. The potato and radish specimens were sectioned into 0.3 cm thick slices using sterile techniques. The leaves were excised from the cabbage, while the remaining stalks were cut into uniform rectangular segments measuring 6 cm × 4 cm. All plant materials underwent a sequential surface sterilization procedure, which involved exposure to 75% (*v*/*v*) ethanol for 60 s. Subsequently, the specimens were thoroughly rinsed three times with sterile distilled water to remove any residual disinfectants.

In this study, *E. coli* DH5α, a non-AHL-degrading strain, served as the negative control. Meanwhile, *B. thuringiensis* B23, which harbors the *aiiA* gene encoding AHL-lactonase and has been confirmed as an AHL-degrading strain [61], and agricultural streptomycin were employed as positive controls. Five experimental treatments were established: (1) pure EC1, (2) EC1 co-inoculated with *E. coli* DH5α, (3) EC1 combined with *B. thuringiensis* B23, (4) EC1 supplemented with agricultural streptomycin, (5) EC1 mixed with XJ-10. Bacterial suspensions (OD_600_ = 1.0 in PBS) of DH5α, B23, XJ-10, and EC1 were triple-inoculated on host plant tissues. All treated sections were incubated in controlled climate chambers at 28 °C for 48 h. The maceration area, quantitatively assessed to evaluate disease progression, was calculated from the measured diameter of the lesion and expressed in square centimeters. The entire biocontrol experiment was independently repeated three times.

### 2.9. Evaluating the Biocontrol Efficacy of Acinetobacter schindleri XJ-10 Crude Enzymes

Crude enzyme extracts from strain XJ-10 were prepared following the methods outlined by [56,64]. The XJ-10 culture was propagated in 10 mL of broth under shaking conditions (200 rpm) at 30 °C. After cells were pelleted via refrigerated centrifugation (10,000× *g*, 10 min, 4 °C), the supernatant containing extracellular enzymes was collected. Harvested cells were washed three times, reconstituted in fresh PBS, and subjected to cellular disruption at 4 °C. The homogenate was centrifuged again (10,000 rpm, 10 min, 4 °C), and the resulting supernatant was collected as the intracellular enzyme extract. To evaluate the efficacy of these crude enzymes against potato soft rot, four treatment groups were established: EC1 alone, EC1 + intracellular enzymes, EC1 + extracellular enzymes, and sterile water control. All treatments were conducted using surface-sterilized potato slices (0.3 cm thick) with four biological replicates. Disease severity was quantified by measuring the area (cm^2^). The entire biocontrol experiment was independently repeated three times.

### 2.10. Acylase Activity Assay

The AHL-degrading enzyme derived from strain XJ-10 was evaluated for acylase activity using a commercial enzymatic assay kit (Beijing Solarbio Science & Technology Co., Ltd., Beijing, China) by spectrophotometric measurement. Strain XJ-10 was cultured overnight in LB medium, after which intracellular and extracellular enzyme fractions were collected separately using established protocols. Four experimental groups were designed for both enzyme fractions: (1) intracellular enzyme treatment, (2) extracellular enzyme treatment, (3) enzyme-free reagent (negative control), and (4) distilled water (blank control).

The enzymatic assay functions via acetyl-CoA-dependent transacetylation to butanol, accompanied by the reduction of 5,5′-dithiobis-(2-nitrobenzoic acid) (DTNB), which produces 2-nitro-5-thiobenzoic acid (TNB). TNB displays a distinct yellow coloration with a maximum absorbance at 412 nm, allowing the spectrophotometric quantification of acylase activity [56,64].

### 2.11. Statistical Analysis

Statistical analyses were performed using GraphPad Prism 10.0 with one-way ANOVA followed by Bonferroni’s post-hoc test. The completely randomized design employed a significance level of α = 0.05, with all biological replicates (*n* ≥ 3) processed independently.

## 3. Results

### 3.1. Isolation, Screening, and Identification of Strain XJ-10

A total of 18 potential QQ strains were isolated from environmental specimens through enrichment culture techniques, with AHL serving as the exclusive carbon source. Primary screening using the *A. tumefaciens* NT1 biosensor system identified strain XJ-10 as exhibiting superior AHL degradation activity (Figure S1), which was subsequently prioritized for mechanistic characterization. This strain demonstrated rapid AHL catabolism, and its QQ capacity was quantitatively validated through high-performance liquid chromatography (HPLC) analyses. The XJ-10 strain has been deposited in the China Center for Type Culture Collection (CCTCC) under the accession number CCTCC M 2017650 at Wuhan University, Wuhan, China.

The morphological and physiological characteristics of strain XJ-10 were systematically analyzed. Cultured on LB agar plates for 48 h, the isolate formed dome-shaped colonies with a smooth texture, opaque appearance, and entire margins (Figure S2A). Distinct beige pigmentation was observed on blood agar plates (Figure S2B). In LB broth, aerobic growth at 30 °C yielded uniform turbidity, consistent with planktonic proliferation. Scanning electron microscopy (SEM) revealed that strain XJ-10 cells were pleomorphic, ranging from short rods to coccobacilli, with a monotrichous flagellar arrangement (Figure S2C). The biochemical characterization of strain XJ-10 is shown in Table S1. Physiochemical profiling demonstrated positive results for catalase activity but negative results for oxidase production, β-hemolysis, gelatinase secretion, and amylolytic capacity (Table S1). The strain exhibited mesophilic growth within 20–41 °C (optimum: 30 °C) and a neutrophilic preference (pH 7.0).

**Figure 1:**
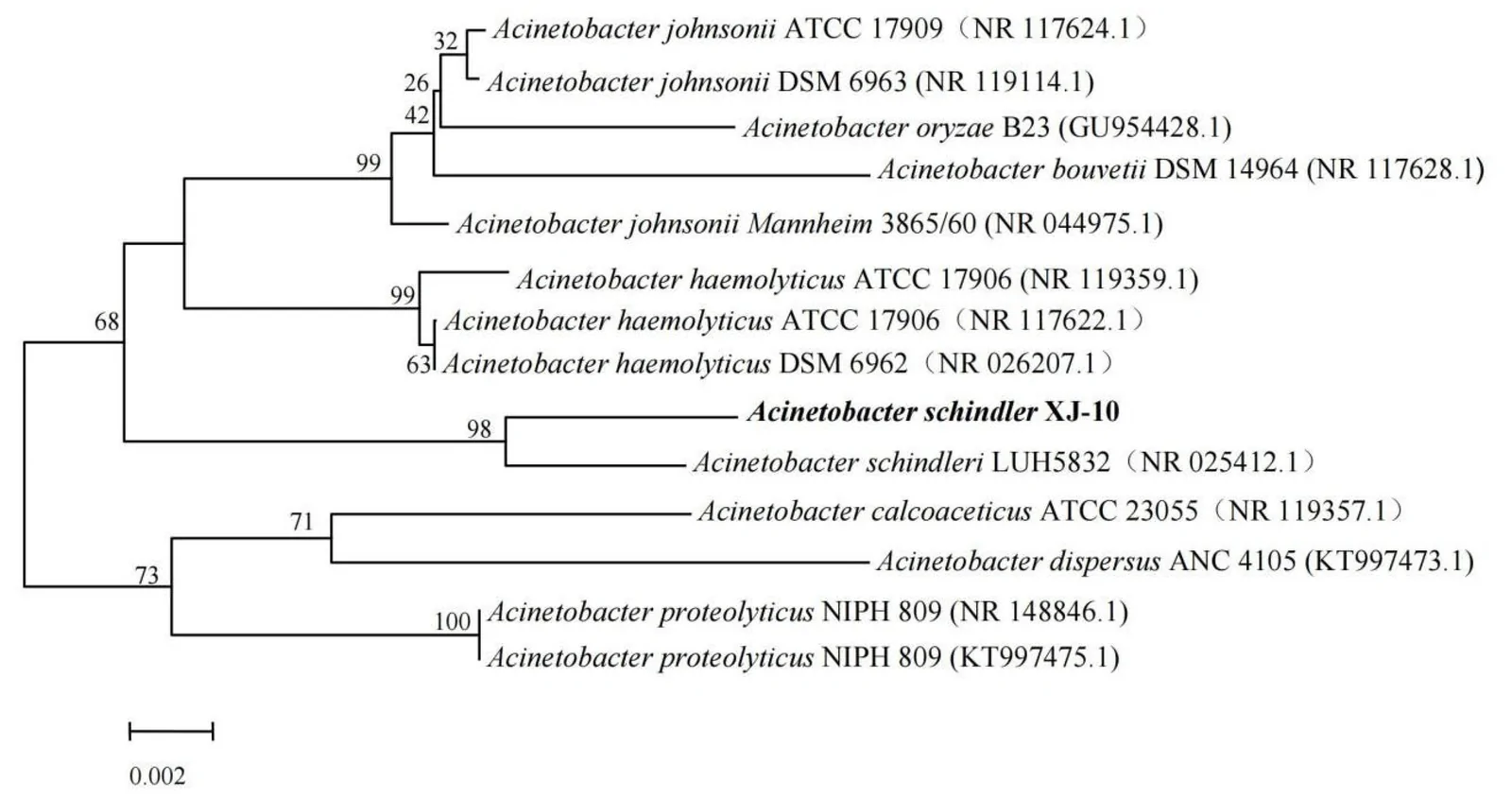
Phylogenetic reconstruction of *Acinetobacter schindleri* XJ-10 based on 16S rDNA gene sequence analysis. The neighbor-joining phylogenetic tree was generated using MEGA 6.0 with 1000 bootstrap replications. Reference strains are labeled with GenBank accession numbers in parentheses. Bootstrap support values are shown at branch nodes. Evolutionary distances are represented by the scale bar (nucleotide substitutions per site).

Genomic DNA of *A. schindleri* strain XJ-10 was amplified using universal bacterial primers, yielding a 1420-bp 16S rDNA gene sequence. BLASTn analysis against the NCBI database revealed 98% sequence similarity to *A. schindleri* LUH5832 (NR025412.1). Phylogenetic reconstruction was performed using the neighbor-joining method in MEGA 6.0 (1000 bootstrap replicates) with type strains from BLAST homology results, positioning XJ-10 within the *A. schindleri* clade (Figure 1). Based on the comprehensive analysis of morphological features, physiological and biochemical characteristics, and 16S rDNA gene sequence, strain XJ-10 was identified as *Acinetobacter schindleri*.

**Figure 2:**
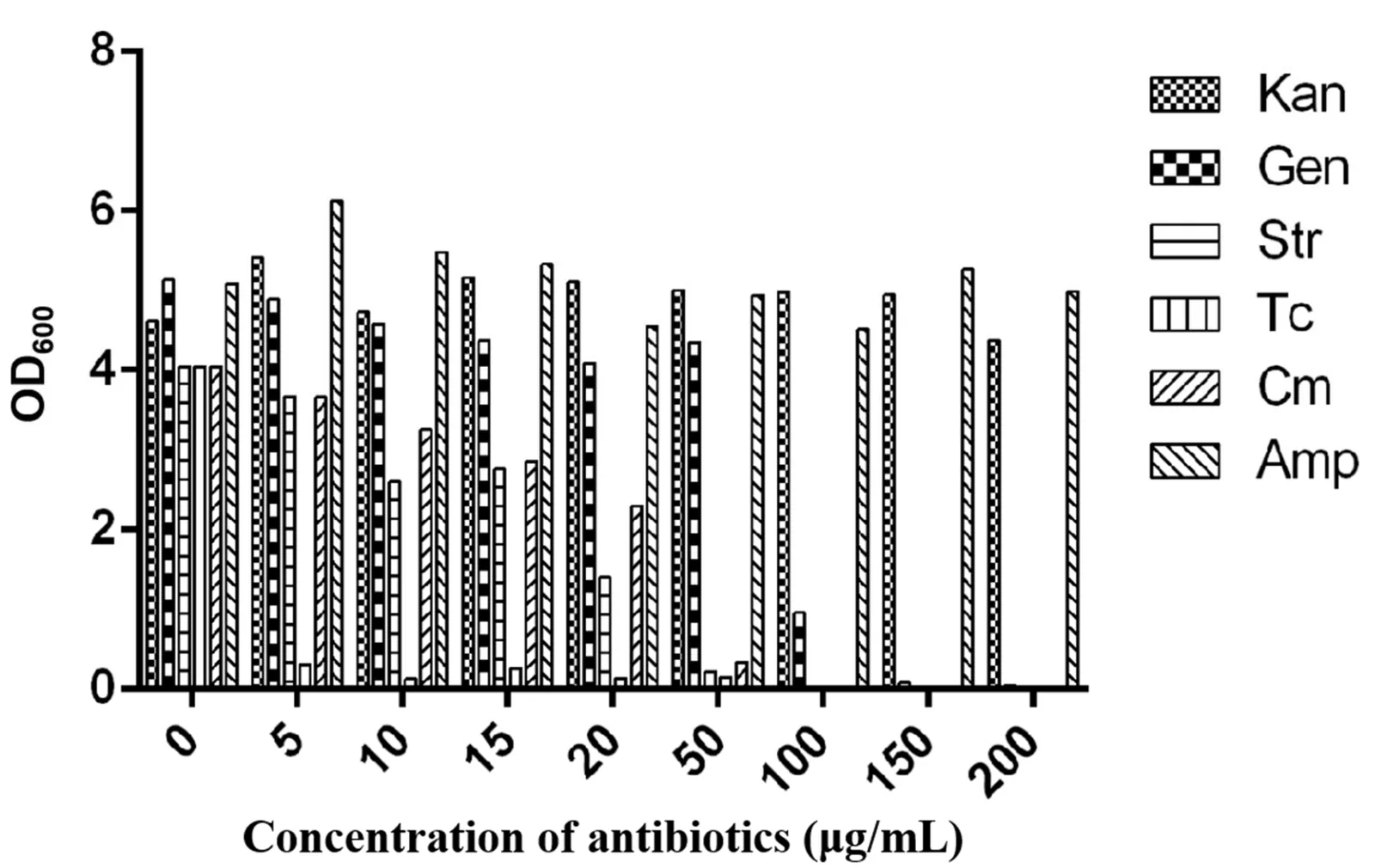
Antimicrobial susceptibility profiling revealed that *Acinetobacter schindleri* XJ-10 exhibited multidrug resistance, demonstrating minimum inhibitory concentrations of ≥400 μg/mL for ampicillin, 200 μg/mL for kanamycin, 100 μg/mL for gentamicin, and 20 μg/mL for both streptomycin and chloramphenicol.

Antimicrobial susceptibility testing of *A. schindleri* strain XJ-10 revealed a multidrug resistance phenotype. The antibiotic susceptibility profile of strain XJ-10 is shown in Figure 2. Strain XJ-10 exhibited resistance to ampicillin at concentrations of 400 μg/mL and above, kanamycin (Kan) at up to 200 μg/mL, gentamicin (Gen) at up to 100 μg/mL, as well as streptomycin (Str) and chloramphenicol (Cm) at concentrations of up to 20 μg/mL.

### 3.2. AHL Degradation Efficiency

**Figure 3:**
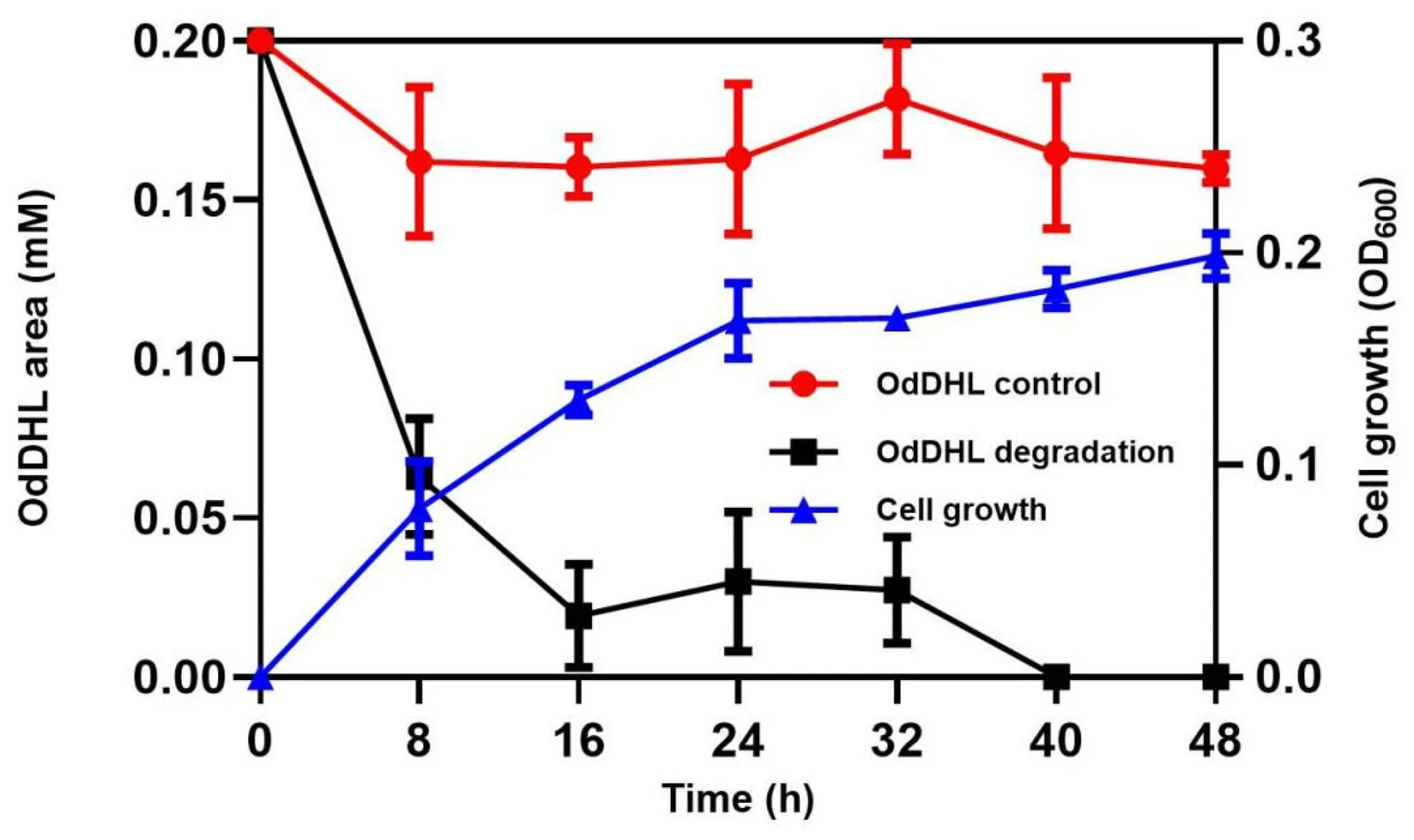
Biodegradation dynamics of *N*-(3-oxododecanoyl)-L-homoserine lactone (OdDHL) during the cultivation of *Acinetobacter schindleri* XJ-10 in minimal salts medium (MSM). Biological triplicates demonstrated substrate depletion kinetics, with complete mineralization achieved within 40 h under optimal conditions (30 °C, 200 rpm). Error bars denote the standard deviation from biological replicates. Experimental groups were differentiated by marker morphology: solid circles (untreated OdDHL), squares (substrate degradation kinetics), and triangles (bacterial growth curves).

The degradation of OdDHL by *A. schindleri* XJ-10 was examined in MSM within controlled laboratory settings (30 °C, 200 rpm). Strain XJ-10 effectively used 0.2 mmol/L OdDHL as its exclusive carbon source. Residual substrate levels were measured by HPLC at multiple time points (0, 8, 16, 24, 32, 40, and 48 h) to monitor the degradation kinetics, with representative chromatograms at 0, 16, and 24 h shown in Figure S3. Uninoculated controls (Figure S3A) exhibited minimal abiotic degradation at 24h, while inoculated cultures achieved 85.71% degradation at 12 h (OD_600_ = 0.144) and 98.80% degradation at 24 h (OD_600_ = 0.233) (Figure S3B,C). Degradation kinetic analysis (Figure 3) revealed a biphasic pattern in degradation activity. The degradation rate of strain XJ-10 peaked during the exponential growth phase (32–40 h), with complete OdDHL degradation achieved within 40 h. During this phase, bacterial biomass increased synchronously, and degradation efficiency was highly correlated with the growth curve. In contrast, the control experiment showed only 20.10% natural degradation of OdDHL within 48 h in sterile MSM, significantly lower than the 100% degradation observed in the strain-treated group at 40 h.

### 3.3. Degradation Products and Pathways of Strain XJ-10

**Figure 4:**
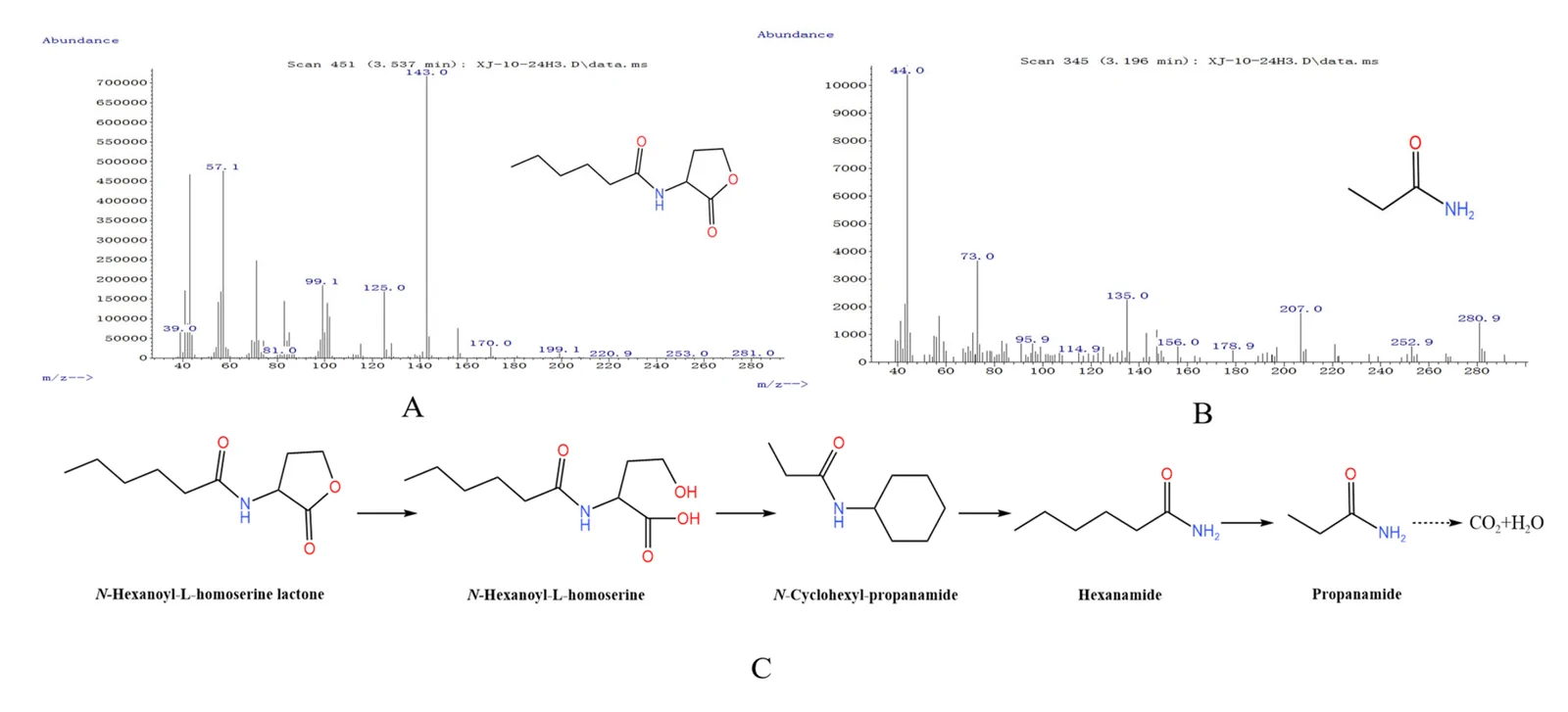
MS-based analysis of AHL degradation products by Acinetobacter schindleri XJ-10. Comparative analysis of degradation products against reference standards from the National Institute of Standards and Technology (NIST) database: (**A**) N-Hexanoyl-L-homoserine lactone; (**B**) propanamide. (**C**) Proposed catabolic pathway of *N*-acyl-homoserine lactone (AHL) in *Acinetobacter schindleri* XJ-10. The process is initiated by hydrolysis of the ester ring, yielding *N*-hexanoyl-L-homoserine as the primary intermediate. This intermediate subsequently undergoes cleavage of the carbon–nitrogen bond to form *N*-cyclohexyl-propanamide. The resulting *N*-cyclohexyl-propanamide undergoes amide bond cleavage, yielding hexanamide and propanamide as transient intermediates. Ultimately, AHL is completely mineralized into carbon dioxide and water.

To elucidate the AHL degradation pathway, *A. schindleri* XJ-10 was cultured in MSM supplemented with 1 mmol/L AHL. To simultaneously detect both the parent AHL molecule and its transient degradation intermediates, samples were harvested at 8, 16, and 24 h during the degradation process. Subsequently, the samples underwent methanol extraction and GC-MS analysis. Key degradation products were identified by matching retention times (RTs) and fragmentation patterns to reference spectra in the NIST library database (Figure S4). A dominant metabolite detected at RT = 3.537 min across all time points exhibited a molecular ion at *m*/*z* 143.0, identified as *N*-hexanoyl-L-homoserine lactone (Figure 4A and Figure S4A). A secondary product at RT = 3.196 min displayed a protonated ion at *m*/*z* 44.0, corresponding to propanamide (Figure 4B and Figure S4B). This degradation product matches findings reported in prior research [65]. Based on structural analysis and intermediate identification, the metabolic pathway of AHL in strain XJ-10 was proposed (Figure 4C). Among the proposed intermediates, only *N*-hexanoyl-L-homoserine lactone and propanamide were experimentally detected by GC-MS [65]. The degradation of AHL begins with the hydrolysis of its ester ring, yielding *N*-hexanoyl-L-homoserine. This intermediate is subsequently cleaved via the breakage of the carbon–nitrogen bond, producing *N*-cyclohexyl-propanamide. Further degradation results in the formation of hexanamide and propanamide. Ultimately, AHL is completely mineralized into carbon dioxide and water. Strain XJ-10 demonstrates efficient degradation and metabolism of AHL, with no detectable intermediate accumulation observed during the process.

### 3.4. Strain XJ-10 Did Not Show Antagonism Against EC1

Antibacterial activity of *A. schindleri* XJ-10 against *D. zeae* EC1 was assessed via well diffusion assay on LB agar plates. After 24 h of incubation at 30 °C, no inhibitory halos were observed around wells containing XJ-10 cells or metabolites (Figure S5). Similarly, control wells (sterile medium and untreated metabolites) exhibited no growth suppression. These findings conclusively demonstrate that strain XJ-10 lacks direct antagonistic effects against EC1, suggesting its biocontrol potential operates through QQ rather than bactericidal activity.

### 3.5. Biocontrol Potential of Strain XJ-10

**Figure 5:**
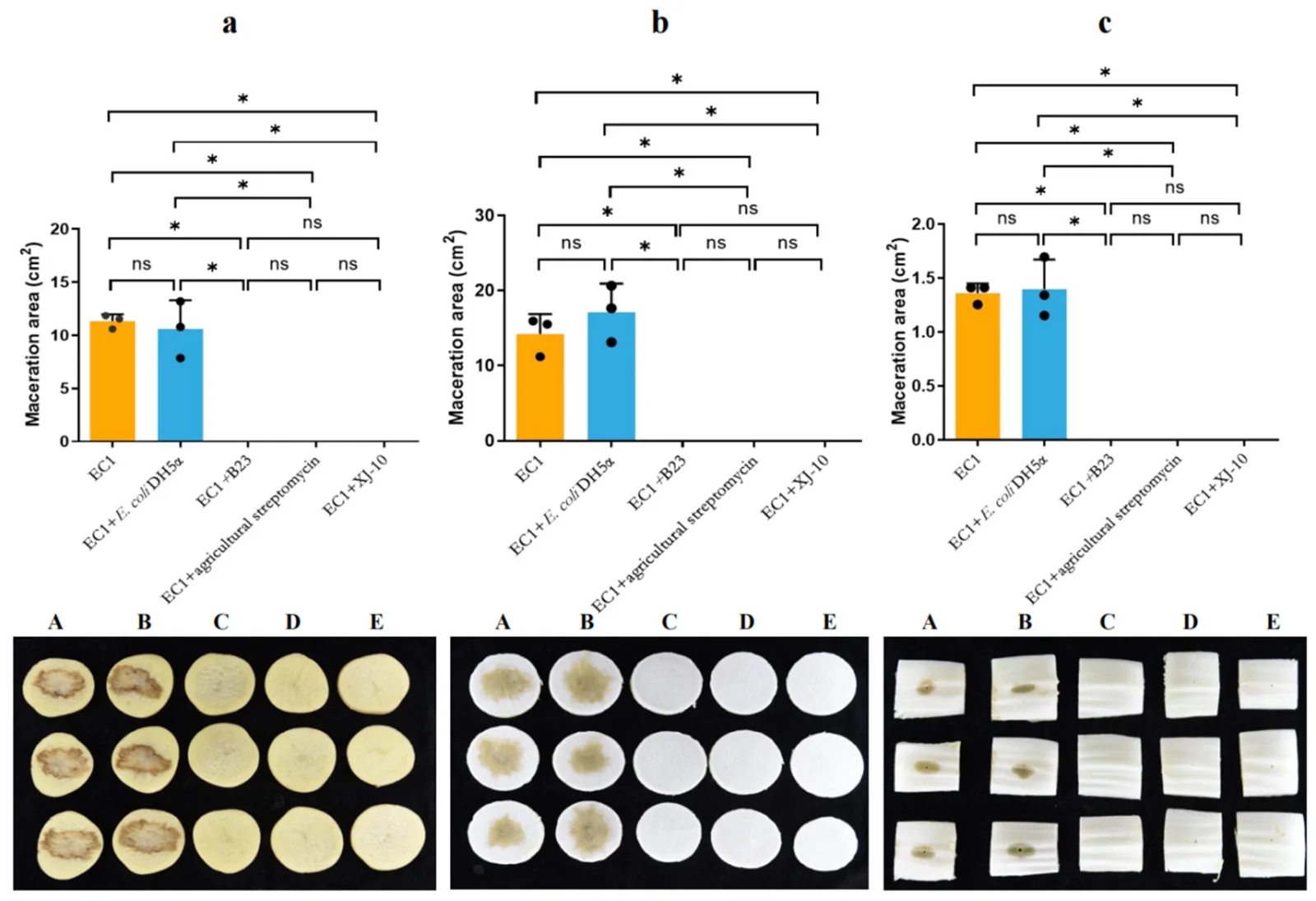
Antagonistic activity of *Acinetobacter schindleri* XJ-10 against *Dickeya zeae* EC1 in plant infiltration assays. (**a**) *Solanum tuberosum* (potato); (**b**) *Raphanus sativus* (radish); (**c**) *Brassica rapa* subsp. *pekinensis* (Chinese cabbage). Treatment groups: (**A**) shows plant tissue inoculated only with *D. zeae* EC1; (**B**) shows plant tissue co-inoculated with EC1 and *E. coli* DH5α; (**C**) shows plant tissue co-inoculated with EC1 and *B. thuringiensis* B23; (**D**) shows plant tissue inoculated with EC1 supplemented with agricultural streptomycin; (**E**) shows plant tissue co-inoculated with EC1 and *A. schindleri* XJ-10. Data were analyzed via one-way ANOVA with Tukey’s multiple comparison test (GraphPad Prism 10.0), adopting a significance threshold of *p* < 0.05. Results are presented as mean ± SD (standard deviation) based on three separate experimental replicates. Significance was denoted by asterisks (*, *p* < 0.05) or “ns” (non-significant, *p* ≥ 0.05).

The biocontrol efficacy of *A. schindleri* XJ-10 against *D. zeae* EC1-induced soft rot was evaluated using an in vitro tissue assay. Disease progression was quantified by measuring lesion expansion areas in tissue sections of potato (*Solanum tuberosum*), radish (*Raphanus sativus*), and Chinese cabbage (*Brassica rapa* subsp. *pekinensis*) (Figure 5a–c). Pathogenicity assays confirmed that strains XJ-10, B23, and *E. coli* DH5α were non-pathogenic to potato. In control groups inoculated solely with EC1, all plant tissues exhibited characteristic soft rot symptoms, including expanding water-soaked lesions (Figure 5A). Co-inoculation of XJ-10 with EC1 significantly reduced disease severity in potato tissues compared to EC1-only and DH5α + EC1 treatments (Figure 5E). Equivalent suppression levels were observed in plant tissues exposed to either EC1 and B23 co-cultures or conventional streptomycin treatments. Strain XJ-10 demonstrated marked biocontrol efficacy, reducing decayed tissue areas across all tested plant species relative to EC1 monoculture. The combined non-pathogenicity and consistent biocontrol efficacy position XJ-10 as a sustainable alternative to chemical controls for *D. zeae*-associated soft rot management.

### 3.6. Biocontrol Effect of XJ-10 Crude Enzymes

**Figure 6:**
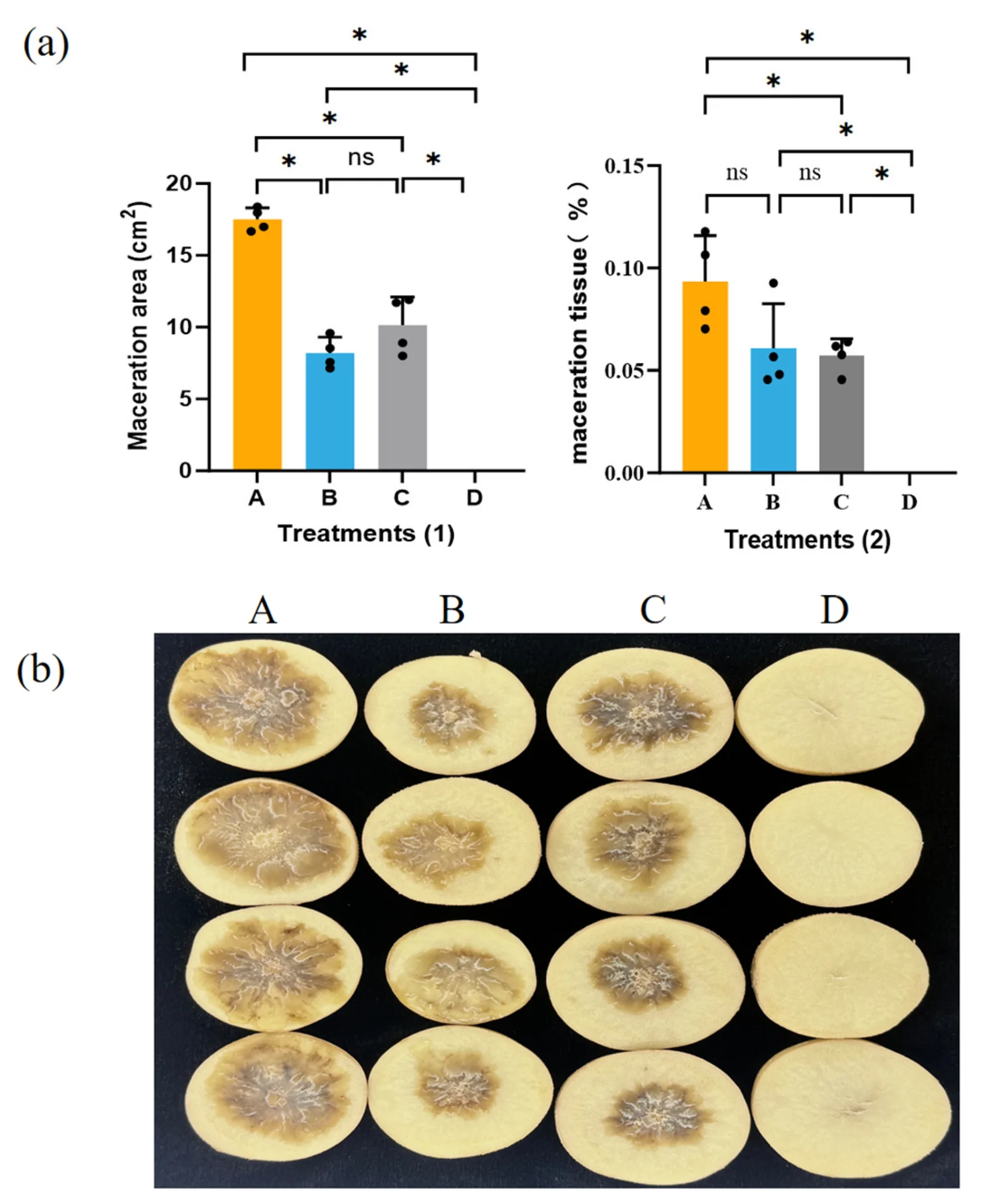
Biocontrol efficacy of *Acinetobacter schindleri* XJ-10 crude enzymes against *Dickeya zeae* EC1. (**a**) Maceration area (1) and maceration tissue (2) across experimental groups. (**b**) Pathogenicity assay on potato tuber sections: (**A**): EC1 inoculation alone; (**B**): EC1 co-inoculated with intracellular enzymes; (**C**): EC1 co-inoculated with extracellular enzymes; (**D**): sterile water control. The experiment utilized a completely randomized design with four biological repeats. Statistical analyses were performed using one-way ANOVA followed by Tukey’s multiple comparison test in GraphPad Prism (version 10.0), with statistical significance set at *p* < 0.05. Data are expressed as mean ± standard deviation (SD) from four independent replicates. Significance levels are indicated by asterisks (*, *p* < 0.05) or “ns” (not significant, *p* ≥ 0.05).

The biocontrol potential of crude enzymes derived from strain XJ-10 was evaluated using an in vitro tissue maceration assay. Potato tuber sections were co-inoculated with *D. zeae* EC1 and XJ-10-derived crude enzymes (intracellular and extracellular fractions), followed by 48 h incubation (Figure 6). The results revealed a significant reduction in the maceration area and maceration tissue percentage in enzyme-treated groups compared to EC1-only controls. Comparative analysis of enzymatic treatments revealed that extracellular enzyme treatment resulted in a larger maceration area than intracellular enzyme treatment. However, no significant differences were observed in their overall efficacy (*p* ≥ 0.05). Overall, both enzyme fractions effectively reduced disease severity compared to the pathogen-only control. These findings demonstrate that the crude enzyme extracts of XJ-10 exhibit potent biocontrol activity against EC1-induced soft rot.

### 3.7. XJ-10 Does Not Have Acylase Activity

To investigate whether *A. schindleri* XJ-10 degrades AHL through acylase-mediated cleavage of the lactone-ring side chain, intracellular and extracellular enzyme extracts were analyzed using a commercial acylase activity assay kit (Figure S6). Notably, both enzyme-supplemented groups and the negative control exhibited identical chromogenic responses, indicating no detectable hydrolysis of the substrate. These findings confirm the absence of acylase activity in strain XJ-10 under the tested conditions.

## 4. Discussion

QS regulates key bacterial processes, including sporulation initiation, biofilm assembly, virulence modulation, antimicrobial tolerance, and horizontal gene transfer [66]. Antibiotics suppress microbial proliferation and enhance immune-mediated pathogen clearance, serving as cornerstone therapeutics for infectious diseases [67,68,69]. However, antibiotic misuse has driven multidrug resistance emergence, with over 70% of clinical isolates resistant to one or more antibiotic classes and accelerated resistance gene dissemination [70,71]. This synergy between antimicrobial resistance (AMR) and QS has accelerated the development of QQ strategies, offering new paradigms for combating bacterial infections [72,73,74].

QQ represents a promising therapeutic strategy that attenuates bacterial pathogenicity by interfering with QS systems while preserving bacterial viability [75]. This approach disrupts autoinducer synthesis or perception, thereby inhibiting virulence factor expression without imposing growth pressure on pathogens [76,77]. QQ operates through two principal mechanisms: enzymatic degradation of QS signals and competitive inhibition of signal–receptor interactions [78,79]. Lactonases, acylases, and oxidoreductases facilitate early intervention by catalytically degrading AHL signal molecules [78,80]. Comparative analyses demonstrate that enzymatic QQ exhibits superior efficacy and biocompatibility compared to conventional QS inhibitors such as 5-fluorouracil, which exhibit cytotoxic limitations [79,81,82]. This approach enhances host immune clearance through reduced virulence factor production while mitigating evolutionary pressures that drive antibiotic resistance [76]. By specifically targeting pathogenicity rather than bacterial survival, QQ technology offers a novel strategy for managing multidrug-resistant infections, addressing both antimicrobial resistance and treatment-related ecological impacts.

In this study, a previously uncharacterized QQ strain, *A. schindleri* XJ-10, was isolated from agricultural soil and exhibited high-efficiency AHL degradation through a novel metabolic pathway independent of acylase activity. The biocontrol efficacy of this strain was validated across multiple host plants, including potato, radish, and Chinese cabbage, against soft rot disease caused by *D. zeae* EC1. Mechanistically, GC-MS analysis is consistent with strain XJ-10 hydrolyzing the lactone ring of *N*-hexanoyl-L-homoserine lactone, suggesting intrinsic lactonase activity. Since the native QS signal of *D. zeae* EC1, OHHL, shares the same conserved homoserine lactone ring [24], the lactonase activity of XJ-10 is expected to effectively disrupt the pathogen’s QS system, thereby suppressing downstream virulence traits such as the production of plant cell wall-degrading enzymes (e.g., pectinases, cellulases, and proteases), bacterial motility, and biofilm formation. Notably, the absence of acylase activity, combined with the inferred lactone-ring hydrolysis, suggests that strain XJ-10 likely employs a lactonase-type enzyme for AHL degradation, though the specific gene(s) await identification. This limitation will be addressed in future work through genome sequencing and functional knockout analyses. This work represents the first application of *A. schindleri* in plant disease management, highlighting its potential as a promising biocontrol agent. The application of QQ-based biocontrol represents an environmentally friendly alternative to conventional chemical pesticides, aligning with the growing interest in sustainable plant disease management strategies [82]. Future studies should focus on identifying key AHL-degrading genes (e.g., via genome sequencing and knockout analysis). Given that *Acinetobacter* sp. is recognized as an opportunistic pathogen in clinical settings, the biosafety of strain XJ-10 must be thoroughly evaluated before agricultural application. Future toxicological assessments should therefore include acute oral toxicity, phytotoxicity, and environmental persistence, as well as evaluation of its impact on non-target soil microorganisms and monitoring of resistance gene transfer. Regulatory frameworks for microbial biocontrol agents should also be considered to guide the safe deployment of this strain. Additionally, formulation optimization and field efficacy trials are needed to facilitate practical application.

## 5. Conclusions

The biocontrol potential of *A. schindleri* XJ-10 was evaluated against soft rot disease in diverse host plants, including potato, radish, and cabbage. Experimental results demonstrated its significant efficacy in suppressing infections caused by AHL-dependent bacterial pathogens, particularly *D. zeae*. This study establishes the application of *A. schindleri* as a quorum-quenching agent for plant disease management. Strain XJ-10 effectively degrades AHL through a proposed metabolic pathway. No detectable acylase activity was observed in strain XJ-10. Furthermore, the XJ-10 strain and its crude enzyme can serve as a promising biocontrol agent in plant applications, effectively mitigating severe diseases induced by *D. zeae* EC1. Strain XJ-10 holds promise for field application within integrated pest management systems, and future efforts toward formulation development and scalable production will facilitate its commercial translation. However, the genetic characterization and toxicological evaluation of strain XJ-10 need to be further studied in order to enhance the usefulness of this biocontrol agent.

## Data Availability

All the data presented in this paper are contained within the manuscript and Supplementary Materials.

## Supplementary Materials

The following supporting information can be downloaded at https://www.mdpi.com/article/10.3390/plants15162439/s1. Figure S1. OdDHL degradation bioassay using the bioreporter strain *Agrobacterium tumefaciens* NT1. (A) Negative control: OdDHL (20 μmol/L) only. (B) Negative control: *Escherichia coli* DH5α (20 μmol/L). (C) Positive control: *Bacillus thuringiensis* subsp. *israelensis* B23 (20 μmol/L). (D) Experimental group: *Acinetobacter schindleri* XJ-10 (20 μmol/L). Increasing zone clearance diameters (inversely proportional to residual OdDHL concentration) correlate with NT1 colony proliferation, demonstrating concentration-dependent quorum quenching activity. Figure S2. Morphological characterization of *Acinetobacter schindleri* XJ-10. (A) Surface morphology of colonies on LB agar after 48 h of incubation. (B) Reverse pigmentation profile on blood agar. (C) Ultrastructural morphology revealed by scanning electron microscopy (5000× magnification). Figure S3. Time-course HPLC quantification of N-(3-oxododecanoyl)-L-homoserine lactone (OdDHL) biodegradation by Acinetobacter schindleri XJ-10 in minimal salts medium (MSM). (A) Negative control: Abiotic degradation in OdDHL-supplemented MSM. Biological degradation profiles at 16 h (B) and 24 h (C), respectively. Figure S4. Mass spectral comparison of degradation products with reference standards from the National Institute of Standards and Technology (NIST) database. (A): *N*-Hexanoyl-L-homoserine lactone; (B): propanamide. For each compound, the upper panel displays the mass spectrum of the experimental sample, while the lower panel shows the corresponding NIST library reference spectrum. Figure S5. Assessment of antagonistic activity between *Acinetobacter schindleri* XJ-10 and *Dickeya zeae* EC1. (A) Strain XJ-10 cell suspension; (B) strain XJ-10-derived metabolites; (C) acetonitrile solvent control; (D) sterile water negative control. No inhibition zones were observed in co-culture assays between strain XJ-10 and the phytopathogen EC1. Figure S6. Acylase activity assay of *Acinetobacter schindleri* XJ-10. (A) Blank control (distilled water); (B) negative control (reaction mixture without enzyme); (C) intracellular enzyme fraction; (D) extracellular enzyme fraction. The assay detects enzymatic activity via the transfer of acetyl groups from acetyl-CoA to butanol, coupled with the reduction of 5,5′-dithiobis-(2-nitrobenzoic acid) (DTNB) to 2-nitro-5-thiobenzoic acid (TNB). TNB exhibits distinct yellow coloration with maximal absorbance at 412 nm. No significant color development or absorbance shift was observed in XJ-10 enzyme fractions (C,D), confirming the absence of detectable acylase activity in strain XJ-10. Table S1. Physiological and biochemical characterization of strain XJ-10. Table S2. Characterization of carbon source utilization of strain XJ-10.
