# Supplementary material for: Characterization of a Novel Quorum Quencher Acinetobacter schindleri Strain XJ-10: AHL Degradation Capability, Metabolic Pathways and Its Role in Soft Rot Disease Biocontrol
Source: Plants (Basel). 2026 Aug 11;15(16):2439. doi: 10.3390/plants15162439 (PMC13517035; doi:10.3390/plants15162439)
Supplement: Supplementary file 1 [file plants-15-02439-s001.zip › plants-4443461-supplementary.pdf]

Supplementary Materials

Supplementary Figures

A B C D

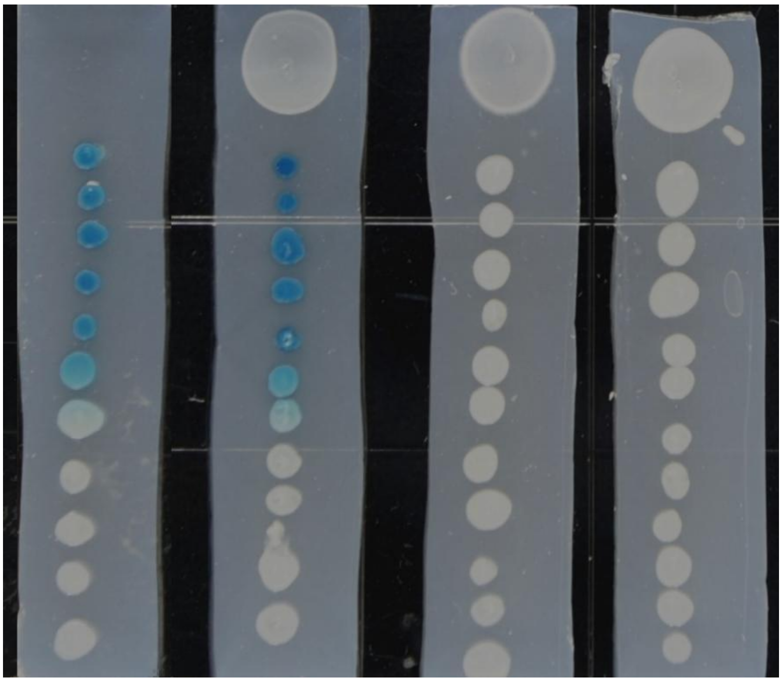

Figure S1. OdDHL degradation bioassay using the bioreporter strain *Agrobacterium tumefaciens* NT1. (A) Negative control: OdDHL (20 μmol/L) only. (B) Negative control: *Escherichia coli* DH5α (20 μmol/L). (C) Positive control: *Bacillus thuringiensis* subsp. *israelensis* B23 (20 μmol/L). (D) Experimental group: *Acinetobacter schindleri* XJ-10 (20 μmol/L). Increasing zone clearance diameters (inversely proportional to residual OdDHL concentration) correlate with NT1 colony proliferation, demonstrating concentration-dependent quorum quenching activity.

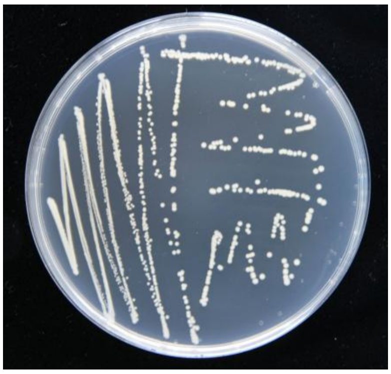

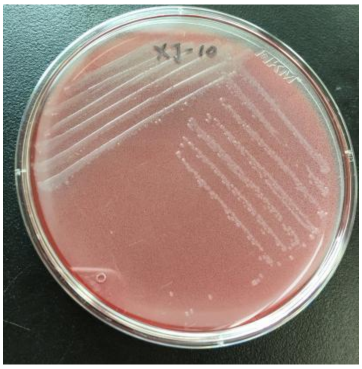

A B

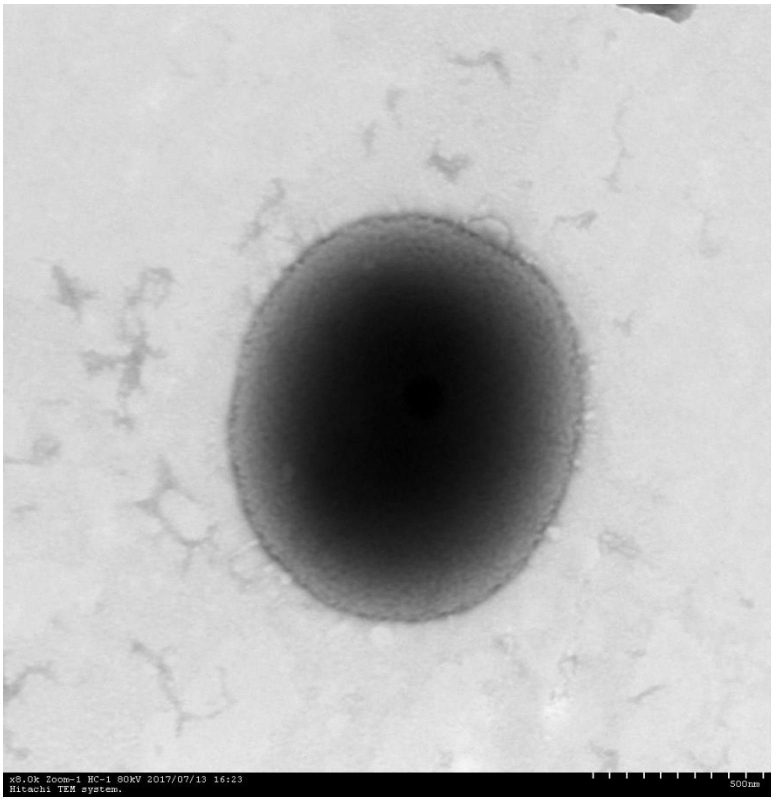

C

Figure S2. Morphological characterization of *Acinetobacter schindleri* XJ-10. (A) Surface morphology of colonies on LB agar after 48 h of incubation. (B) Reverse pigmentation profile on blood agar. (C) Ultrastructural morphology revealed by scanning electron microscopy (5,000 × magnification).

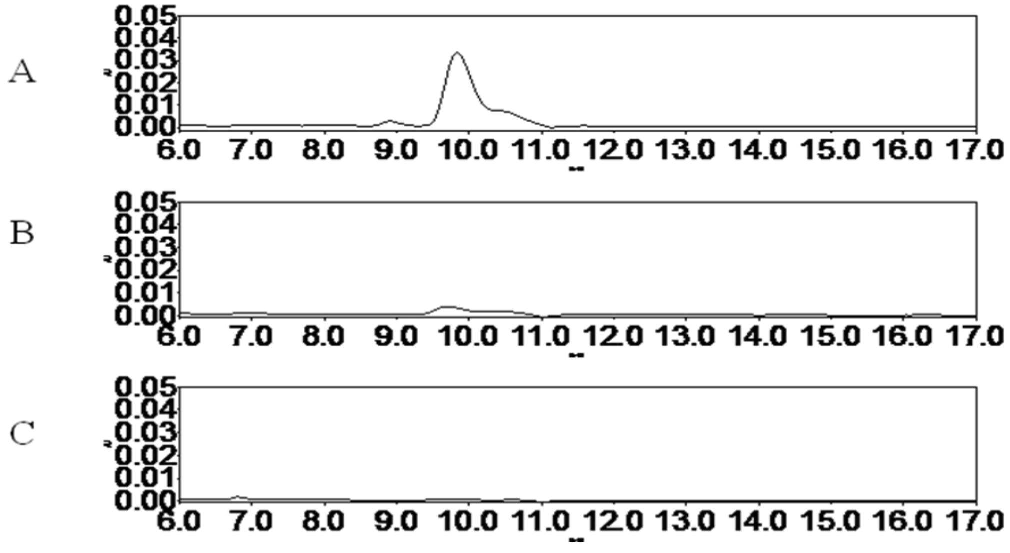

Figure S3. Time-course HPLC quantification of N-(3-oxododecanoyl)-L-homoserine lactone (OdDHL) biodegradation by *Acinetobacter schindleri* XJ-10 in minimal salts medium (MSM). (A) Negative control: abiotic degradation in OdDHL-supplemented MSM. Biological degradation profiles at 16 h (B) and 24 h (C), respectively.

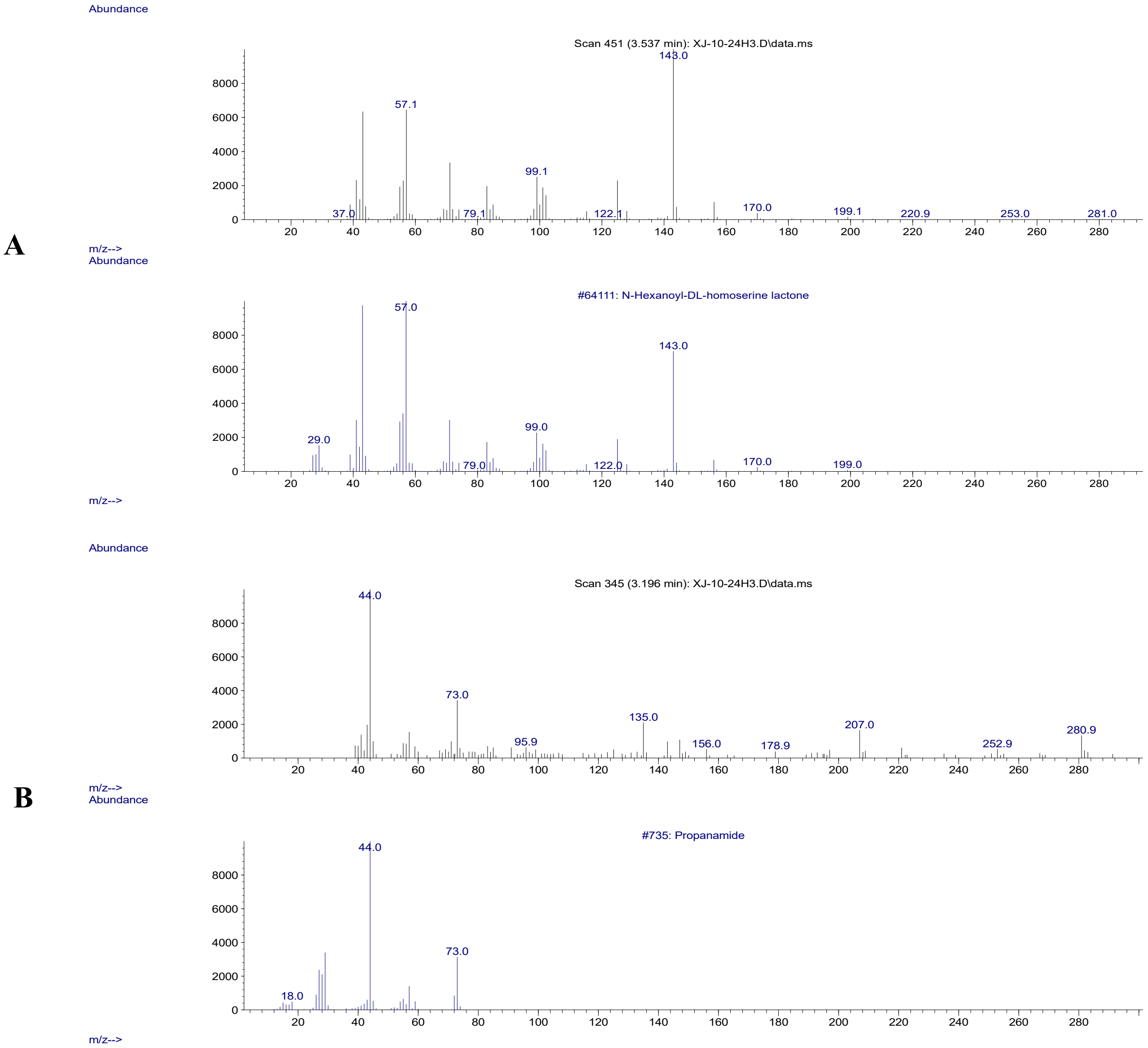

Figure S4. Mass spectral comparison of degradation products with reference standards from the National Institute of Standards and Technology (NIST) database. A: N-Hexanoyl-L-homoserine lactone; B: Propanamide. For each compound, the upper panel displays the mass spectrum of the experimental sample, while the lower panel shows the corresponding NIST library reference spectrum.

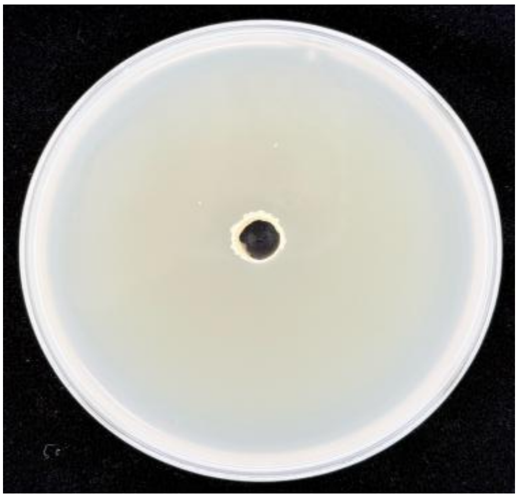

A

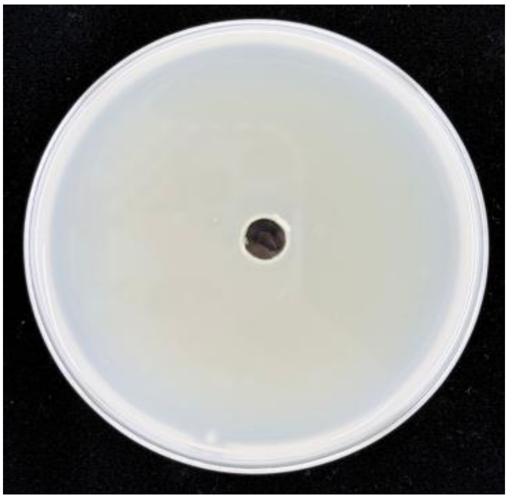

B

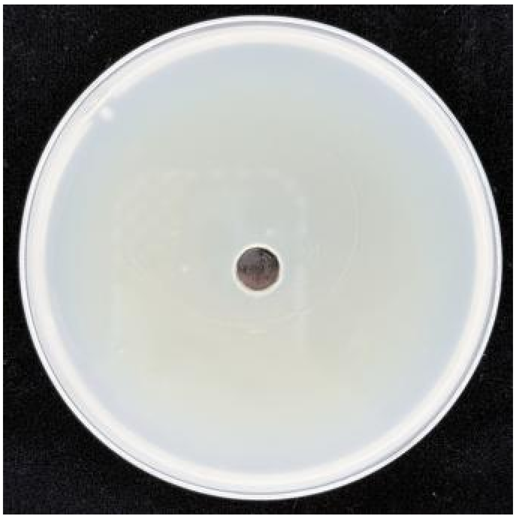

C

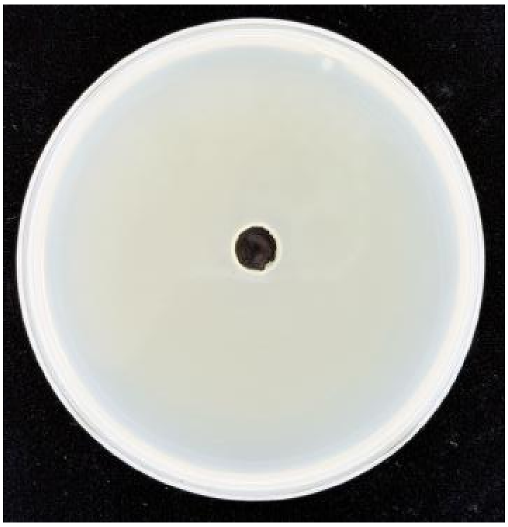

D

Figure S5. Assessment of antagonistic activity between *Acinetobacter schindleri* XJ-10 and *Dickeya zeae* EC1. (A) Strain XJ-10 cell suspension; (B) Strain XJ-10-derived metabolites; (C) Acetonitrile solvent control; (D) Sterile water negative control. No inhibition zones were observed in co-culture assays between strain XJ-10 and the phytopathogen EC1.

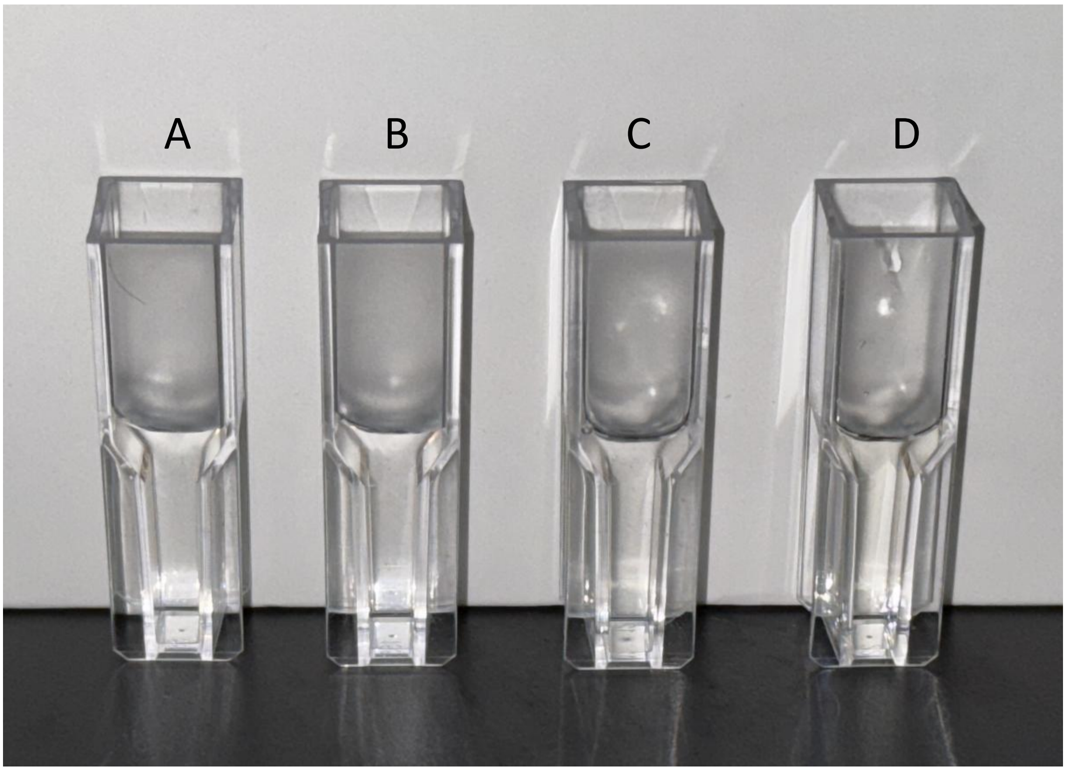

Figure S6. Acylase activity assay of *Acinetobacter schindleri* XJ-10. (A) Blank control (distilled water); (B) Negative control (reaction mixture without enzyme); (C) Intracellular enzyme fraction; (D) Extracellular enzyme fraction. The assay detects enzymatic activity via the transfer of acetyl groups from acetyl-CoA to butanol, coupled with the reduction of 5,5′-dithiobis-(2-nitrobenzoic acid) (DTNB) to 2-nitro-5-thiobenzoic acid (TNB). TNB exhibits distinct yellow coloration with maximal absorbance at 412 nm. No significant color development or absorbance shift was observed in XJ-10 enzyme fractions (C, D), confirming the absence of detectable acylase activity in strain XJ-10.

Supplementary Tables

Table S1 Physiological and biochemical characterization of strain XJ-10

Characteristics	Results	Characteristics	Results
Gram staining	−	Catalase	+
Anaerobic growth	−	Growth at 10°C	−
Catalase	+	Growth at 20°C	+
Oxidase	−	Growth at 25°C	+
Gelatin liquefaction	−	Growth at 30°C	+
Hemolytic activity	−	Growth at 41°C	+
Starch hydrolysis	−	Growth at 44°C	−
Growth at pH 7	+	Growth at pH 6	+
Growth at pH 5	−	Growth in 1% NaCl	+
Growth in 4% NaCl	+	Growth in 8% NaCl	−

Note: "+": Positive utilization; "−": Negative utilization.

Table S2 Characterization of carbon source utilization of strain XJ-10

Carbon Source	Result	Carbon Source	Result
Lithium chloride	Positive	Dextrin	Negative
D-Maltose	Negative	D-Trehalose	Negative
D-Cellobiose	Negative	Gentibiose	Negative
Sucrose	Negative	D-Turanose	Negative
Stachyose	Negative	D-Raffinose	Negative
α-D-Lactose	Negative	D-Melibiose	Negative
β-Methyl-D-glucoside	Negative	D-Salicin	Negative
N-Acetyl-D-glucosamine	Negative	N-Acetyl-β-D-mannosamine	Negative
N-Acetyl-D-galactosamine	Negative	N-Acetylneuraminic acid	Negative
α-D-Glucose	Positive	D-Mannose	Positive
D-Fructose	Negative	D-Galactose	Positive
3-Methylglucose	Positive	D-Fucose	Positive

L-Fucose	Positive	L-Rhamnose	Positive
Inosine	Positive	D-Sorbitol	Negative
D-Mannitol	Negative	D-Arabitol	Negative
myo-Inositol	Negative	Glycerol	Negative
D-Glucose-6-phosphate	Negative	D-Fructose-6-phosphate	Positive
D-Aspartic acid	Negative	D-Serine	Negative
Gelatin	Negative	Glycyl-L-proline	Negative
L-Alanine	Positive	L-Arginine	Negative
L-Aspartic acid	Negative	L-Glutamic acid	Negative
L-Histidine	Negative	L-Pyroglutamic acid	Negative
L-Serine	Negative	Pectin	Negative
D-Galacturonic acid	Positive	L-Galactonic acid γ-lactone	Negative
D-Gluconic acid	Negative	D-Glucuronic acid	Positive
Glucuronamide	Positive	Mucic acid	Positive
Quinic acid	Positive	Saccharic acid	Positive
p-Hydroxy-phenylacetic acid	Negative	Methyl pyruvate	Positive

Methyl D-lactate	Positive	L-Lactic acid	Positive
Citric acid	Negative	α-Keto-glutaric acid	Negative
D-Malic acid	Negative	L-Malic acid	Positive
Aztreonam	Positive	Tween 40	Positive
Potassium tellurite	Positive	α-Hydroxy-butyric acid	Positive
β-Hydroxy-D,L-butyric acid	Positive	α-Keto-butyric acid	Positive
Acetoacetic acid	Positive	Propionic acid	Positive
Acetic acid	Positive	Formic acid	Negative
Sodium lactate	Positive	Fusidic acid	Negative
Sodium butyrate	Positive	Troleandomycin	Negative
Rifamycin	Positive	Minocycline	Negative
Sodium bromate	Negative	Guanidine hydrochloride	Positive
Sodium tetradecyl sulfate	Positive	Vancomycin	Positive
Tetrazolium violet	Positive	Tetrazolium blue	Positive
Nalidixic acid	Positive		
